# Altering the Fluorination Anodization Pathway of Silicon Near an Electrolyte Freezing Point Promotes the Formation of Blue-Photoluminescent Microstructures

**DOI:** 10.1021/acsami.5c24266

**Published:** 2026-01-29

**Authors:** Yu-Sheng Chiou, Chao-Chia Cheng, Ryan Wen-Shuo Li, Benjamin Tien-Hsi Lee

**Affiliations:** † Department of Mechanical Engineering, 34911 National Central University, Taoyuan city 320317, Taiwan, Republic of China; ‡ Department of Physics, National Central University, Taoyuan city 320317, Taiwan, Republic of China

**Keywords:** cryogenic treatment, fluorination, nanostructure, silicon photonics, photoluminescence (PL)

## Abstract

A fluoride-anion (F^–^) governed electrochemical etching process, i.e., fluorination anodization, is used to construct unique silicon nanostructures owing to its reaction pathway, presenting a powerful and versatile strategy for fabricating advanced microsystems and quantum-based photonics devices. This fluorination anodization approach, which produces nanocrystals in anodized silicon, takes full advantage of the inherent crystalline quality of the prime wafers to achieve well-recognized photoluminescence via the quantum confinement effect. However, for heavily boron-doped (*p* ^*+*^-type) silicon, fluorination anodization fails to produce a photoluminescent layer because the high doping level results in a coarse etching morphology. In contrast, performing anodization with the electrolyte precooled near its freezing point rather than at room temperature changes the outcome of UV irradiation on the anodized surface—from forming an optically absorbing black layer to producing a bright blue layer composed of nanocrystals (1.8–2.2 nm). Moreover, the cryogenically treated etching behavior appears to shift from anisotropic to isotropic as a result of the altered interfacial reactions. This transition may be attributed to the suppression of crystallographic etching by oxidation–etching control rather than Gösele–Lehmann–model–etching control. The cryogenically designed fluorination pathway near the electrolyte freezing point significantly influences the anodization process of heavily boron-doped silicon, thereby enabling the formation of surface microstructures suitable for low-resistivity silicon photonic and quantum devices. Overall, we report a physical cryogenic treatment that alters the interfacial reactions during fluorination anodization by operating the electrolyte near its freezing point. Under these cryogenic conditions, the anodization behavior is substantially altered, thereby facilitating the formation of hydrogenated or fluorinated surface nanostructures on silicon and promoting advanced semiconductor and photonics manufacturing.

## Graphical abstract

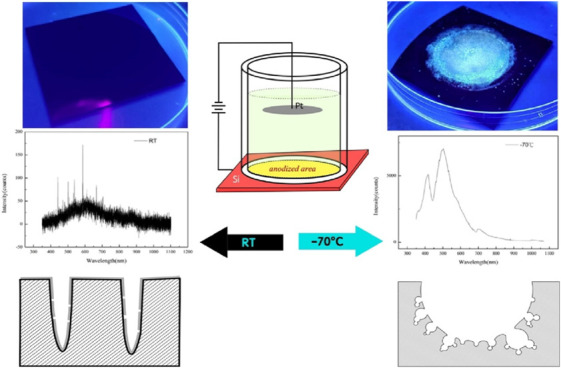

## Introduction

The quantum confinement effect caused by nanostructuring silicon^1^ has inspired and advanced multiple technological eras in edge-cutting photonics and electronics, such as those related to 5*G*/6G communication,^2^ quantum-confined Si tunnel FETs,^3^ single-electron transistors (SETs),^4,5^ silicon quantum-dot lasers,^6,7^ and quantum computing.^8^ To obtain silicon nanocrystals of the highest quality comparable to that of industrial prime wafers, the top-down etching approach remains the most successful method. The resulting nanocrystals are higher quality than those produced by conventional bottom-up methods such as CVD, PVD, and ion implantation.^9,10^ In the early 1990s, Prof. Canham et al. reported that quantum confinement in porous silicon^11,12^ formed by fluorination anodization could overcome this fundamental limitation of the indirect bandgap. By exposing the electrochemically anodized regular *p*-type silicon to ultraviolet (UV) irradiation, visible red photoluminescence was observed—a landmark discovery that initiated the field of silicon photonics. Prof. Ulrich Gösele et al. proposed the Gösele–Lehmann model,^13,14^ a well-established framework describing the formation of porous silicon, which serves as the technological basis for silicon nanostructuring. The model emphasizes the role of fluoride anions (F^–^) in promoting pore evolution through hole-mediated electrochemical dissolution and has guided the optimization of etching conditions favorable for porous silicon formation. However, it may not account for the possible involvement of electrolyte cations during the initial stages of silicon dissolution.^15^

Moreover, nanoscale *p*-type porous silicon exhibits superior electro-optic properties owing to the presence of dense and fine silicon nanocrystals within the anodized layer, which can be produced through inhibition induced by laser irradiation.^16,17^ The photoluminescence of the anodized or porous silicon layer is a key characteristic of optoelectronic devices, and the advancement of silicon photonics^18^ is particularly highlighted in this study.

On the basis of the IUPAC classification, porous silicon is categorized by pore size into three types: microporous (<2 nm), mesoporous (2–50 nm), and macroporous (>50 nm).^19^ The pore size and distribution directly influence the wall thickness and morphology, which in turn control the size of the silicon nanocrystals confined by the etching framework. In other words, the nanocrystal size primarily depends on both the type and concentration of dopants in the silicon substrate, which in turn influence PL generation. Several studies^20–22^ have reported that PL and silicon nanocrystals frequently appear in microporous silicon formed through the anodization of lightly doped *p*-type substrates, with red PL being a characteristic observation.^23,24^

In contrast, under identical electrochemical conditions, fluorination anodization of heavily boron-doped *p* ^+^-type silicon typically produces mesoporous structures, usually resulting in a black, velvet-like surface that exhibits strong light absorption but no detectable PL.^25–27^ Conversely, numerous studies have attempted to induce PL in heavily boron-doped silicon to leverage its superior electronic properties. A common strategy involves postprocessing the porous silicon to reduce the pore dimensions and facilitate nanocrystal formation. Typically, oxidized porous silicon undergoes selective etching^28^ through buffered hydrofluoric acid (BHF) cycles, progressively shrinking the crystal grains to the nanometer scale and enabling PL activation. Moreover, effective surface passivation is crucial for achieving strong and stable PL, as it minimizes the density of dangling bonds and surface defects that serve as nonradiative recombination centers.

The blue PL generated through oxidation is often transient, typically lasting only a few hours.^29^ In contrast, our current study revealed that the blue PL directly generated via cryogenic fluorination anodization remains stable for more than one year. We attribute this remarkable stability to direct surface passivation achieved through hydrogen termination during HF treatment.^13^ The quantum confinement effect enhances the ability of surface dangling bonds to capture hydrogen ions,^30^ which is consistent with the behavior observed in wafer bonding,^31^ thereby maintaining this passivation and sustaining the PL emission from nanostructured silicon. Furthermore, the restricted etching activity of fluoride ions on silicon oxide at near-freezing temperatures may facilitate the formation of an oxyfluoride (*SiO*_*x*_ *F*_*y*_) surface layer via fluorine retention within the oxide matrix. This structure may act as a kinetic barrier against moisture permeation and subsequent oxidation in ambient air,^32^ allowing the blue PL to persist for more than 15 months.

The steps and mechanism^13,19,33,34^ of porous silicon formation through electrochemical fluorination can be generalized by the following chemical equations, as shown below:

1 $${\mathrm{Si}}_{\left(s\right)}{+\mathrm{2HF}}_{\left(\mathrm{aq}\right)}+\left(\mathrm{2‐}\alpha \right)h^{+}\to {\mathrm{SiF}}_{2(s)}+{2H^{+}}_{\left(\mathrm{aq}\right)}{+\alpha e}^{‐}$$

2 $${\mathrm{SiF}}_{2(s)}{+\mathrm{2HF}}_{\left(\mathrm{aq}\right)}\to {\mathrm{SiF}}_{4(s)}{+H}_{2(g)}$$

3 $${\mathrm{SiF}}_{4(s)}{+\mathrm{2HF}}_{\left(\mathrm{aq}\right)}\to H_{2}{\mathrm{SiF}}_{6(\mathrm{aq})}$$

In eq 1, *h* ^+^represents an electronic hole injected under the applied anodic bias, which drives the oxidation of surface silicon atoms and initiates the subsequent fluorination steps. These interfacial reactions describe the stepwise fluorination and dissolution of silicon during anodization in HF-based electrolytes, in which surface silicon reacts with HF in the presence of holes to form intermediate and final fluorinated species.

Although the precise electrochemical pathways underlying anodic dissolution during fluorination anodization remain incompletely understood, it is well established that holes are critical for initiating redox reactions at the silicon–electrolyte interface, ultimately driving pore formation. To satisfy the stringent material requirements for next-generation quantum devices, precise size control is indispensable. Since the electronic and optical properties of silicon nanocrystals are strictly governed by their diameter,^35^ achieving ultrasmall sizes (below 3 nm) with precision is a primary prerequisite for strong quantum confinement. Furthermore, enhancing quantum confinement stability often relies on optimizing the surface environment and strain.^36^ Additionally, emerging regulations on nanomaterial toxicity highlight the urgent need for sustainable, heavy-metal-free alternatives,^37^ positioning silicon as an ideal candidate. To address these challenges (size, stability, scalability, and sustainability), in this work, we introduce a simple yet effective physical pathway by which the electrolyte temperature is reduced and maintained near its freezing point (−70 °C to −80 °C), thereby obviously altering the pathway of fluorination anodization. In contrast, laser-assisted suppression is constrained by localized irradiation,^16^ whereas postprocessing approaches such as high-temperature annealing or chemical oxidation exhibit poor precision in regulating nanocrystal dimensions.^38,39^ We report for the first time the direct fabrication of heavily boron-doped porous silicon exhibiting a bright, tunable red-to-blue PL via a cryogenic electrochemical pathway. This achievement, illustrated in Figures 1 and S1 (Supporting Information), highlights the distinct advantages of the cryogenic pathway in fluorination anodization. Notably, cryogenic conditions not only reduce the overall reaction rate but also fundamentally alter the anodization behavior. The size of the nanocrystals within the nanostructured silicon layer can be estimated from the PL spectrum on the basis of the quantum confinement effect. According to the Brus quantum confinement model,^40^ the bandgap energy varies with the nanocrystal radius *R* as follows eq 4:

4 $$E_{\mathrm{gap}}\left(R\right)=E_{\mathrm{bulk}}+\frac{h^{2}\pi^{2}}{2R^{2}}\left(\frac{1}{m_{e}}+\frac{1}{m_{h}}\right)\frac{1.8e^{2}}{4\pi \varepsilon R}+...$$

**Figure 1:**
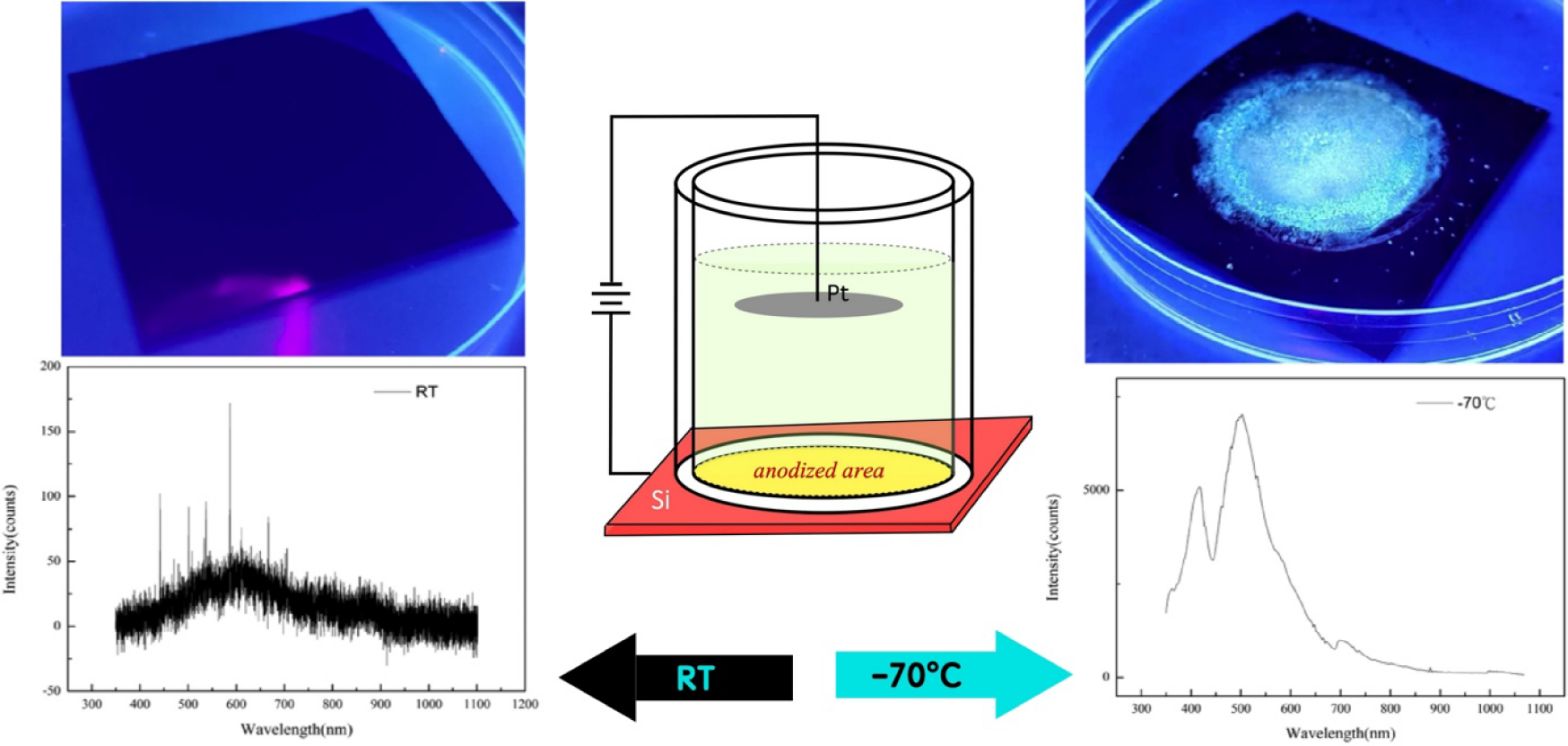
Schematic illustration of the electrochemical fluorination anodization setup and temperature-dependent PL characteristics of heavily boron-doped p^+^-type silicon. Anodization at room temperature (RT, left side) produces a black surface without detectable PL emission. Anodization at −70 °C (right side) results in a surface emitting bright blue light, accompanied by multiple PL peaks at 415 and 500 nm, demonstrating enhanced emission intensity and a shift toward shorter wavelengths. The resulting light-emitting nanostructures are CMOS-compatible and stable at room temperature, offering a promising platform for on-chip integration in silicon photonics and quantum devices.

Furthermore, the PL peak energy can be empirically correlated with the silicon nanocrystal diameter *d* via Pavesi’s eq 5:^17,41^

5 $$E_{PL}^{corr}=1.17+\frac{3.73}{d^{1.39}}+\frac{0.081}{d}-0.245$$

where *d* is the diameter of the nanocrystal (in nanometers).

## Materials and Methods

For the experiments, 6-in. (100) heavily boron-doped CZ silicon prime wafers (Wafer Works Corp., Taiwan) with resistivities of 0.003–0.004 Ω·cm were utilized. The wafers were diced into 5 cm × 5 cm square samples for the experiments (the detailed material parameters are listed in Table S1). The experiments were conducted under two conditions: room-temperature anodization at 25 °C, which served as the reference, and cryogenic anodization down to −80 °C, which was performed with a temperature-controlled electrolyte regulated by liquid nitrogen flow. The cryogenic fluorination anodization experiments were carried out via a custom-built, temperature-controlled system, as illustrated in Figure 2. The key components and experimental procedures are described as follows:

**Figure 2:**
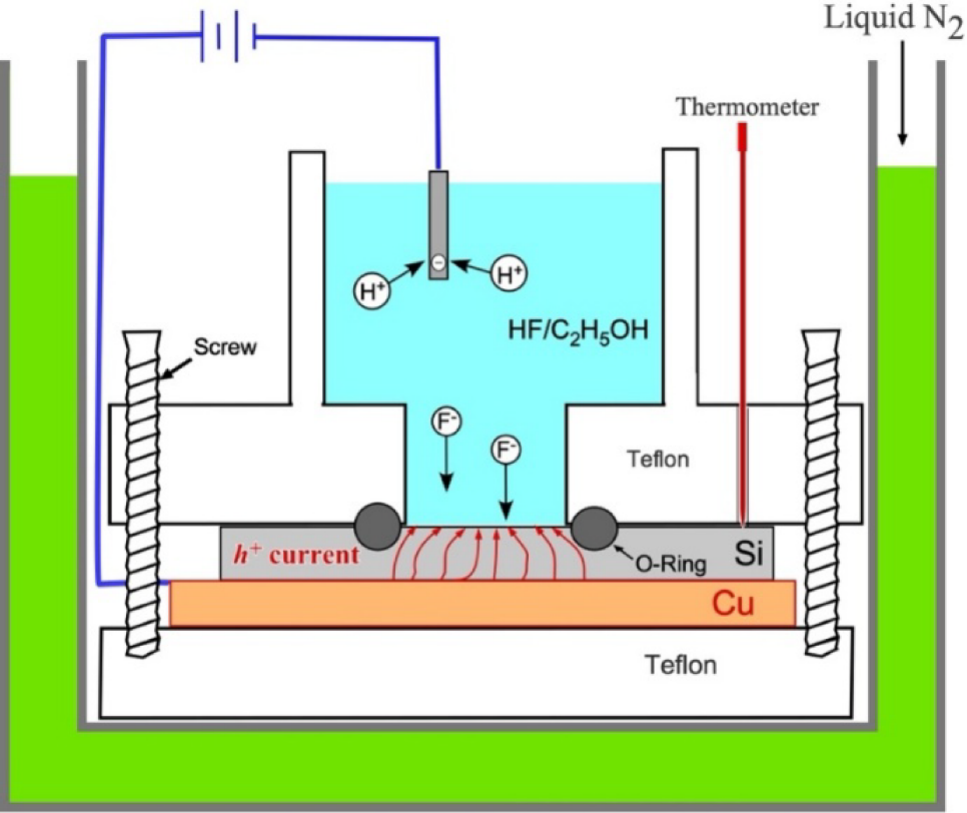
Schematic diagram of the experimental setup for electrochemical anodization at room or cryogenic temperatures. The setup is designed to achieve temperatures of −30 °C to −80 °C via liquid nitrogen. The core setup for anodization consists of a structured arrangement including a PTFE etching bath, an O-ring for sealing, a silicon sample, a copper electrode, a platinum electrode and a bottom support.

### Cooling System and Temperature Control (Figure 2)

a. Setup and Stability: The electrolyte was not merely precooled; the entire Teflon reaction tank was surrounded by a bath of liquid nitrogen throughout the anodization process to maintain a constant temperature (−70 °C to −80 °C). This setup ensured uniform cooling and prevented temperature fluctuations caused by the exothermic nature of the reaction.

b. Temperature monitoring: The temperature was monitored directly via a corrosion-resistant thermocouple immersed in the electrolyte to ensure stability and precise regulation during cryogenic treatment.

### Electrochemical Cell

a. Reaction vessel: The reaction vessel was constructed from Teflon to ensure compatibility with hydrofluoric acid (HF)-based electrolytes.

b. Sealing system: Viton O-rings were employed to maintain a leak-proof environment, which is particularly critical under cryogenic conditions because of thermal contraction.

### Electrolytes and Electrical Parameters

a. Electrolyte composition: The electrolyte is composed of a 1:1 (v/v) mixture of concentrated HF (49%) and ethanol (99%). In addition to balancing reactivity and viscosity, ethanol acts as a reliable antifreezing agent, lowering the freezing point of the mixture to approximately −78 °C.^42^ This freezing point is well below our operation temperature of −70 °C, effectively preventing any ice formation during the process.

b. Current control: A constant hole (*h* ^*+*^ *)* current was applied to drive the anodization process, with adjustments made to compensate for temperature-dependent charge transport effects. For representative samples in this study, a current of 300 mA was applied for 60 min at a specific controlled temperature.

### Experimental Procedure

Precooling: The electrochemical cell was precooled to the target temperature (−70 °C to −80 °C) using LN_2_.
Electrolyte introduction: The electrolyte was then introduced under controlled conditions to prevent premature freezing.
Anodization: Anodization was carried out under a constant current, while voltage–time (*V*–*t*) profiles were recorded to monitor the reaction kinetics.

After processing, the samples were rinsed with cold ethanol to preserve the surface morphology.

Thermal uniformity: The setup minimized temperature gradients across the Si/electrolyte interface.
Interfacial stability: The system maintained consistent reaction conditions despite cryogenic challenges such as changes in electrolyte viscosity.
Reproducibility: Multiple trials were conducted to verify the reliability of both the cooling and electrochemical parameters.

This methodology enables a systematic investigation of temperature-dependent fluorination mechanisms, bridging the gap between conventional anodization and cryogenic quantum nanostructure formation. The exposed surface area of each substrate in contact with the electrolyte was 9.62 cm^2^. The electrolyte used for anodization was a freshly prepared 1:1 (v/v) mixture of 48 wt % HF and 95 wt % ethanol, both of which were CMOS grade. A calibrated thermocouple was employed to ensure precise temperature monitoring and control throughout the process.

## Results and Discussion

The PL spectra and visible emission colors of heavily boron-doped *p* ^+^-type silicon anodized at room temperature and at a cryogenic temperature of −70 °C are compared in Figure 3a. All the measurements were conducted under identical excitation powers and integration times to ensure direct comparability. As shown in Figure 3a, no observable PL emission is detected from the sample anodized at room temperature (25 °C), indicating a lack of luminescent nanostructure formation under standard conditions. In contrast, during fluorination anodization at −70 °C, the anodized surfaces presented distinct PL signatures characterized by two dominant emission peaks at 415 nm (violet) and 500 nm (blue). This result is clearly visible as cyan–blue luminescence, which is indicative of quantum-confined emission enabled by enhanced nanoscale structuring at cryogenic temperatures. We compare the PL spectra of a sample measured at −70 °C in December 2023 and remeasured after more than one year of ambient air storage in March 2025. Remarkably, despite the absence of surface passivation or vacuum protection, the PL intensity remains largely stable, with only the violet emission band exhibiting a notable reduction in intensity. These results confirm that cryogenically anodized heavily boron-doped silicon retains its quantum-confined PL characteristics with minimal degradation over extended ambient exposure. The preserved spectral profile and broad visible emission further underscore its potential for long-term integration in photonic and quantum applications. These temperature-dependent PL characteristics (Figure S2 in the Supporting Information) support the hypothesis that cryogenic fluorination anodization induces a transition in etching behavior and nanostructure formation, thereby enabling spectral tunability from red to blue through thermal modulation alone. This exceptional stability is attributed to the robust surface passivation layer formed during the cryogenic process. The surface chemical composition of the silicon samples was analyzed via high-resolution X-ray photoelectron spectroscopy (XPS) to elucidate the mechanistic differences between room temperature (RT) and cryogenic processing. The RT sample (Figure 3e) exhibits a significantly higher O 1 s intensity (2.2 × 10^5^ cps) than the −70 °C sample (Figure 3d) (8.0 × 10^4^ cps), which is attributed to disordered postetching oxidation occurring on the high-surface-area structures. Furthermore, the F 1 s spectra revealed a stark contrast in fluorine retention between the two regimes. The RT sample (Figure 3g) shows minimal fluorine intensity (∼4.6 × 10^3^ cps), which is consistent with the fluorination–etching (Gösele–Lehmann) model, where silicon dissolves rapidly into soluble *SiF*_*x*_ species.

**Figure 3:**
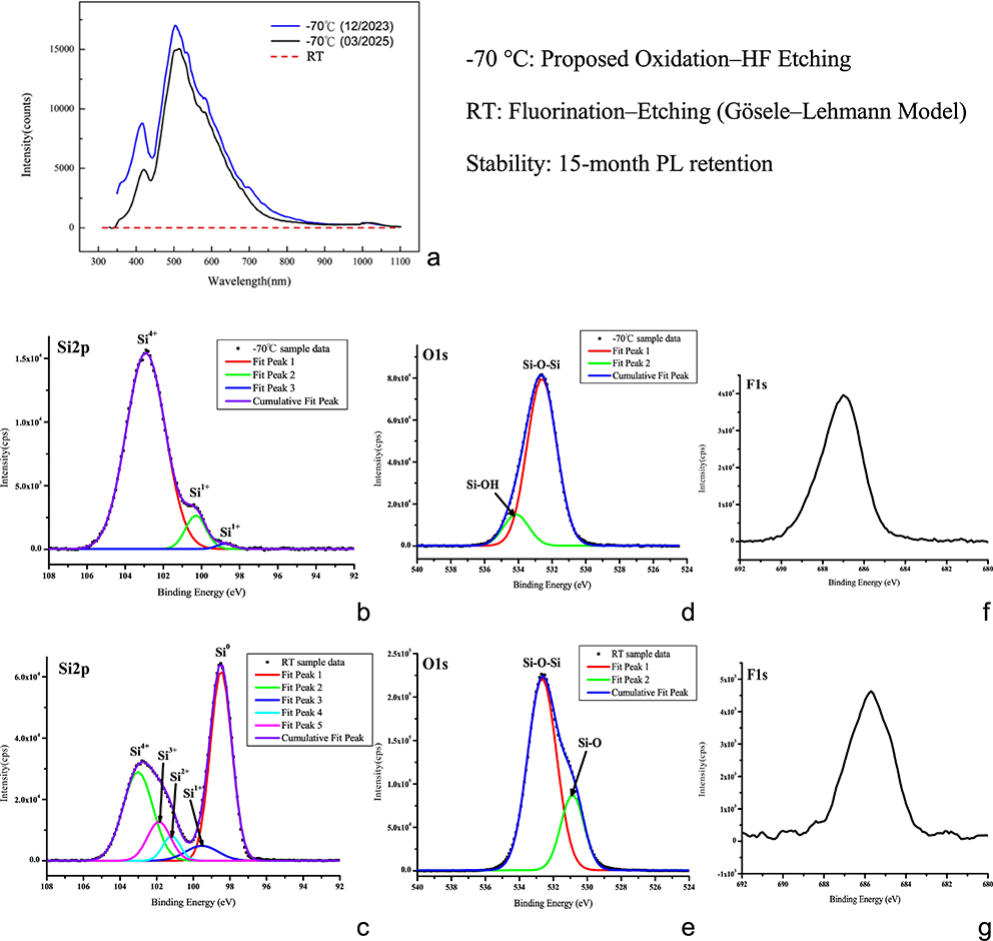
Correlation between optical emission, surface chemical state, and long-term stability. (a) PL spectra comparing the nonemissive RT sample with the blue-emissive −70 °C sample. The −70 °C sample demonstrated near-perfect spectral retention after 15 months of ambient storage (Dec 2023 vs Mar 2025). (b–g) High-resolution XPS spectra for Si 2p, O 1 s, and F 1 s. Mechanism Transition: The dominance of *Si* ^0^ at RT (c) confirms a standard fluorination-etching path, whereas the shift to *Si^4+^* at −70 °C (b) indicates an oxidation-governed regime. To demonstrate the effects of surface passivation, the RT sample (e) exhibits a significantly higher O 1 s intensity (2.2 × 10^5^ cps) than the −70 °C sample (d) (8.0 × 10^4^ cps), which is attributed to disordered postetching oxidation occurring on the unpassivated surface. In contrast, the −70 °C sample (f) exhibited a dramatic 10-fold increase in F 1 s intensity (4 × 10^4^ cps) compared with that of the RT sample (g) (5 × 10^3^ cps) and a binding energy shift to 687.0 eV, indicating that fluorine was integrated into a surface chemical matrix. These data, coupled with the *Si^4+^* dominance observed in Figure 3b, strongly suggest the formation of an in situ passivation *SiO_x_F_y_* shell. These results suggest that the *SiO_x_F_y_* shell formed at −70 °C may act as a kinetic diffusion barrier that stabilizes the blue luminescent nanocrystals.

In contrast, the −70 °C sample (Figure 3f) exhibits a dramatic 10-fold increase in F 1 s intensity (∼4.0 × 10^4^ cps) and a binding energy shift to 687.0 eV, indicating that fluorine has been doped into a stable chemical matrix rather than remaining a transient reactant. These findings, coupled with the dominant *Si* ^*4+*^ signature observed in the Si 2p spectrum at −70 °C (Figure 3b), strongly suggest a transition to an oxidation–HF etching pathway. We propose that this cryogenic process facilitates the in situ formation of a stable superhydrophobic fluorinated silica (oxyfluoride, *SiO*_*x*_ *F*_*y*_) shell.^32^ The superhydrophobic character of this formed oxyfluoride shell plays a critical role in the long term stabilization of the emissive structures. As suggested by molecular dynamics simulations, the doping of fluorine significantly lowers the surface interaction energy with water molecules. This hydrophobic nature effectively repels ambient moisture, thereby establishing a kinetic diffusion barrier that prevents oxidative degradation of the silicon nanocrystals. This facilitates the exceptional 15-month photoluminescence (PL) stability observed exclusively in the −70 °C samples (Dec 2023 vs Mar 2025), as shown in Figure 3a, whereas the RT reference remains nonemissive due to the absence of quantum-confined nanocrystals within its coarse morphology (Figure 4b).

**Figure 4:**
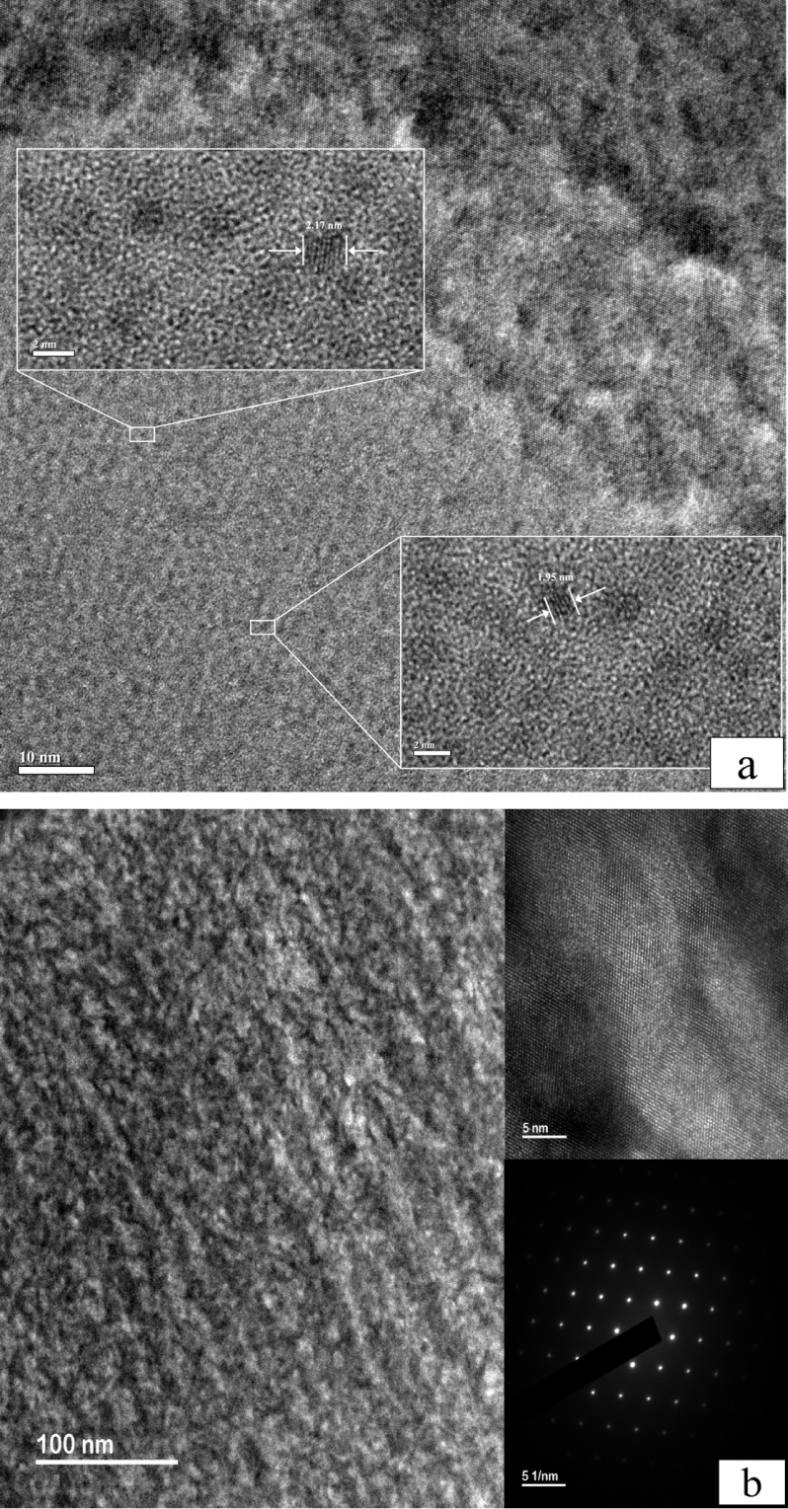
Comparative TEM analysis of *p^++^*-type silicon anodized under cryogenic and room-temperature conditions. (a) Cryogenic Anodization (−70 °C): Cross-sectional view (scale bar = 10 nm) revealing isolated, ultrasmall nanocrystals. High-resolution insets highlight lattice fringes of individual crystals with diameters of 2.17 nm (upper) and 1.95 nm (lower-right), respectively. (b) Room-Temperature Anodization (25 °C): Characterization of a control sample etched at 300 mA for 60 min. The morphology consists of a continuous mesoporous network devoid of quantum-confined nanocrystals. The high-resolution inset (upper right, scale bar = 5 nm) displays well-defined silicon crystalline domains exceeding 14 nm in width. The corresponding Selected Area Electron Diffraction (SAED) pattern (lower right, scale bar = 5 1/nm) exhibits sharp, discrete spots, verifying the bulk-like single-crystal integrity of the framework remaining after aggressive ambient-temperature etching.

To elucidate the microstructural origin of the observed PL, transmission electron microscopy was performed on silicon samples anodized at −70 °C, as shown in Figure 4. A cross-sectional analysis of the sample anodized at −70 °C reveals ultrasmall nanocrystals with diameters in the range of 1.9–2.2 nm. These dimensions fall within the regime where quantum confinement effects become significant. In accordance with the empirical relationship reported by Ledoux et al.^43^ and Pavesi,^41^ the PL wavelength is directly correlated with the nanocrystal size: blue emission at 500 nm corresponds to ∼2.2 nm nanocrystals, and violet emission at 415 nm corresponds to ∼1.9 nm nanocrystals. This correlation between the nanocrystal size and PL wavelength suggests that the PL observed in cryogenically anodized silicon is likely governed by quantum confinement effects. This correlation highlights that the silicon nanocrystal size, and hence its photoluminescence wavelength, can be precisely modulated by adjusting the anodization temperature within the cryogenic regime. In contrast, the room-temperature (RT) control sample shows non-PL emission under UV irradiation. Instead, the RT silicon framework consists of a continuous mesoporous network composed of large crystalline domains. High-resolution TEM image of this RT structure reveals a consistent lattice with a measured width of 15.49 nm (Figure 4b), representing a bulk-like silicon skeleton. The corresponding selected area electron diffraction (SAED) pattern displays sharp, discrete spots, further confirming that the silicon remains a high-quality single crystal rather than being fragmented into quantum-confined dots. Although a previous research^44^ has indicated that nanocrystals may form in room-temperature porous silicon, the resulting silicon microstructures typically exhibit a broad size distribution, extending up to 25 nm, which is attributed to their inherent fragility and continuous mesoporous morphology. In our study, the absence of blue photoluminescence in the room-temperature samples is attributed to these crystallite sizes far exceeding the quantum confinement threshold (∼3 nm) required for blue emission. This contrast underscores the vital role of the cryogenic pathway in achieving the precise size control (1.8–2.2 nm) necessary for the observed optoelectronic properties.

According to the Gösele–Lehmann model, at room temperature, fluoride anions (F^–^) generated from HF dissociation preferentially attach to silicon dangling bonds, with hole-assisted cleavage of Si–Si covalent bonds leading to the formation of volatile SiF_4(s)_, which subsequently dissolve into the electrolyte through complexation with HF molecules to form soluble species such as H_2_SiF_6(aq)_. This room-temperature electrochemical etching mechanism is inherently anisotropic and governed by surface energetics and crystallographic orientation owing to the atomic packing density.^33^ In contrast, under cryogenic conditions, anodization still occurs, but the resulting profile exhibits isotropic characteristics, as shown in Figure 5, which are distinct from the anisotropic features observed during fluorination at room temperature.

**Figure 5:**
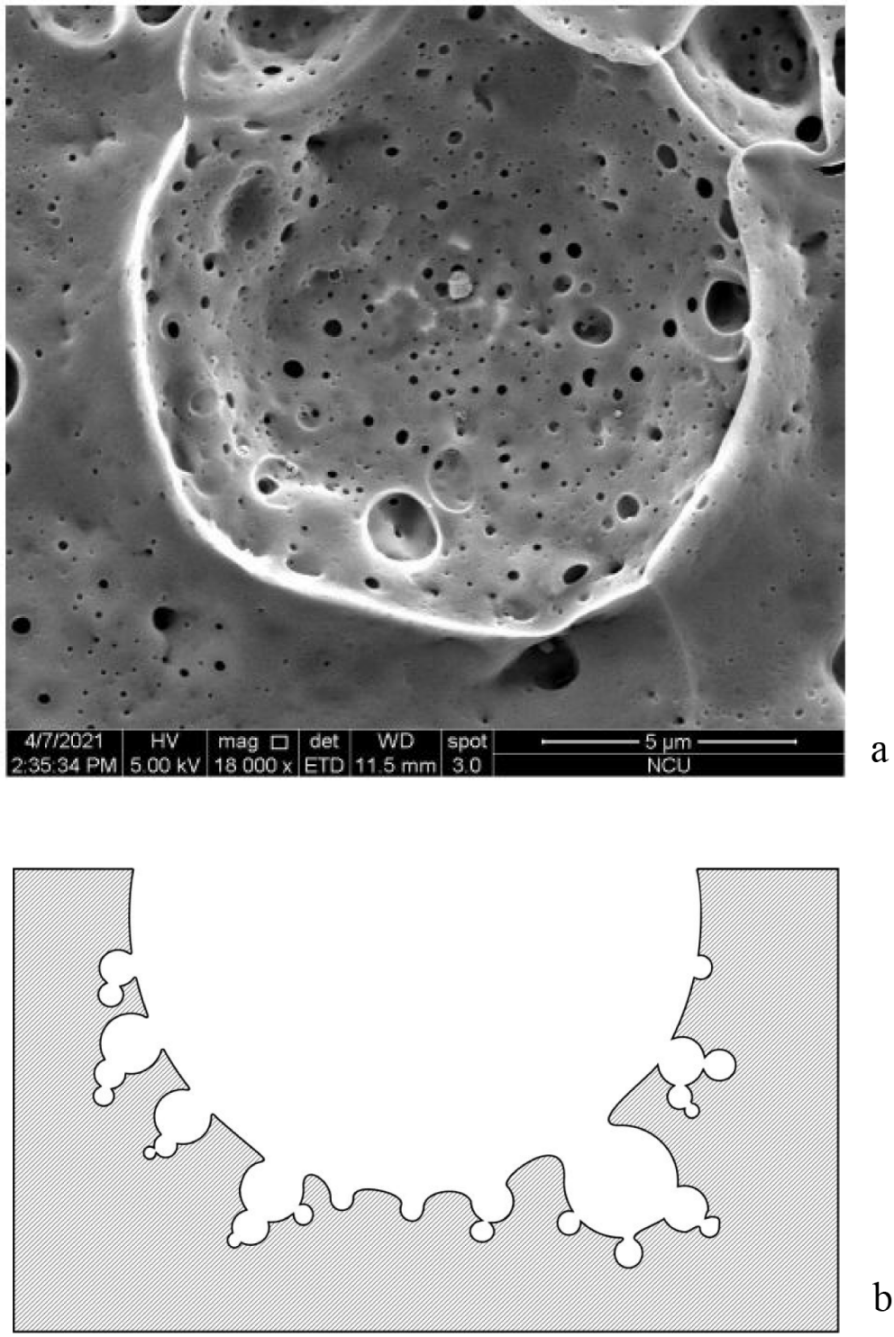
Top-view SEM image of silicon anodized at −80 °C. (a) The surface has a highly isotropic morphology characterized by uniformly distributed circular pits and rounded pore openings. The smooth and spherical-like profiles indicate an isotropic etching response that differs markedly from the anisotropic, crystal orientation governed dissolution typically observed at room temperature. (b) Schematic cross-section illustrating that the isotropic etching behavior is confined to localized regions where the fluoride-anion concentration remains sufficiently high to sustain the reaction, rather than occurring uniformly across the entire surface.

### Anodization Mechanism Under Cryogenic Conditions

At cryogenic temperatures, HF molecules at sufficiently high concentrations rarely dissociate,^45^ resulting in a negligible concentration of fluoride anions; under such conditions, silicon may, in principle, undergo negligible etching. However, anodization (electrochemical etching) still occurred, but the etching became isotropic rather than anisotropic, as observed at room temperature. To explain the observed isotropic electrochemical etching, we propose the following etching mechanism, which is based on two working hypotheses. When a sufficiently high bias was applied under cryogenic conditions, hole injection promoted the in situ redox reaction of silicon with water molecules to form SiO_2_ rather than SiF_4_ (Gösele–Lehmann model). Second, the HF molecules undergo dissociation driven by the energy released from the exothermic formation of SiO_2_,^46^ yielding fluorine anions (F^–^) and protons (H^+^), causing SiO_2_ dissociation. This second reaction breaks Si–O–Si bonds and results in the formation of soluble hexafluorosilicic acid (H_2_SiF_6_).

The overall reaction can be described by chemical reaction eqs 6 and 7:^47^

6 $${\mathrm{Si}+2h}^{+}{+\mathrm{2OH}}^{‐}\to {\mathrm{SiO}}_{2}{+H}_{2}$$

7 $${\mathrm{SiO}}_{2}+\mathrm{6HF}\to H_{2}{\mathrm{SiF}}_{6}{+2H}_{2}O$$

The in situ oxidation–driven etching behavior may be evidenced by the continuous evolution of gas bubbles during anodization (see Videos S1–S4). This etching pathway contrasts with the anisotropic morphologies typically observed at room temperature under the Gösele–Lehmann framework, in which fluoride-anion-mediated dissolution is strongly dictated by crystallographic orientation.^48^ In terms of the changes in enthalpy of the competing reactions, the in situ oxidation pathway becomes thermodynamically favorable over the fluorination reaction as the electrolyte temperature approaches the freezing point.^15^

The speciation of HF in aqueous solution, as inferred from water-activity measurements, is strongly temperature dependent, with the neutral HF molecule becoming the predominant species at lower temperatures and higher acid concentrations.^49^ In addition, the temperature dependence of acid dissociation equilibria is well established and can be described by the van’t Hoff relationship, which predicts a decrease in the acid dissociation constant, Ka, of weak acids as the temperature decreases.^50^ Consistent with the endothermic nature of HF dissociation in water, both the Ka and the activity of free fluoride ions decrease as the temperature decreases. Although Ka values at −70 °C have not been reported, measurements of hydrogen-ion activity (pH) and freezing-point depression in HF-H_2_O mixtures further substantiate the strong thermodynamic sensitivity of HF speciation to temperature.^49,51^ Therefore, under our cryogenic and highly saturated electrolyte conditions, the availability of free F^–^ may become even more restricted, providing a thermodynamic basis for the predominance of water-derived oxidation pathways over fluoride-mediated reactions. Under such cryogenic conditions, the suppression of F^–^ availability weakens the crystallographic orientation-dependent etching described by the Gösele–Lehmann model, thereby shifting the overall reaction balance toward in situ oxidation-driven dissolution. As a result, the anodization behavior is governed predominantly by in situ oxidation–reaction energetics rather than by fluoride–anion–mediated etching, which is consistent with the isotropic features observed experimentally.

While this in situ oxidation-driven etching model serves as a preliminary framework, the underlying mechanism remains open to discussion, and future investigations may further refine or revise this understanding. This work thus promotes continued exploration and constructive dialogue within the research community dedicated to advancing the field of semiconductor electrochemistry.

To explore the boundaries of this isotropic regime, Figures 5 and S7 (Supporting Information) present top-view SEM images of a sample anodized at −80 °C, which is very challenging near the freezing point of the electrolyte. Under these extreme conditions, anisotropy is completely absent, strongly implying a fundamental change in the underlying fluoride–anion etching mechanism. The top-view and cross-sectional SEM images in Figure 6 reveal significant microstructural evolution as the anodization temperature decreases from room temperature to cryogenic conditions. The porous silicon formed at room temperature exhibited a highly ordered array of uniformly distributed surface pores (Figures 6a and S3 in the Supporting Information) and fully anisotropic, vertically aligned mesoporous columns in the cross section (Figure 6b and Figure S4 in the Supporting Information). These well-ordered columnar structures are characteristic of mesoporous silicon formed in heavily boron-doped silicon under standard electrochemical anodization conditions. Unlike the porous structures observed for anodization at room temperature, the surface anodized at −70 °C or at −80 °C is dominated by circular pit-like formations filled with isotropically developed spherical nanostructures (Figures 6c and Figure S5 in the Supporting Information). The cross-sectional view (Figures 6d and Figure S6 in the Supporting Information) reveals shallow, wide pits devoid of vertical or branched porous silicon, with spherical nanostructures and small holes uniformly covering the pit walls, confirming the loss of anisotropy.

**Figure 6:**
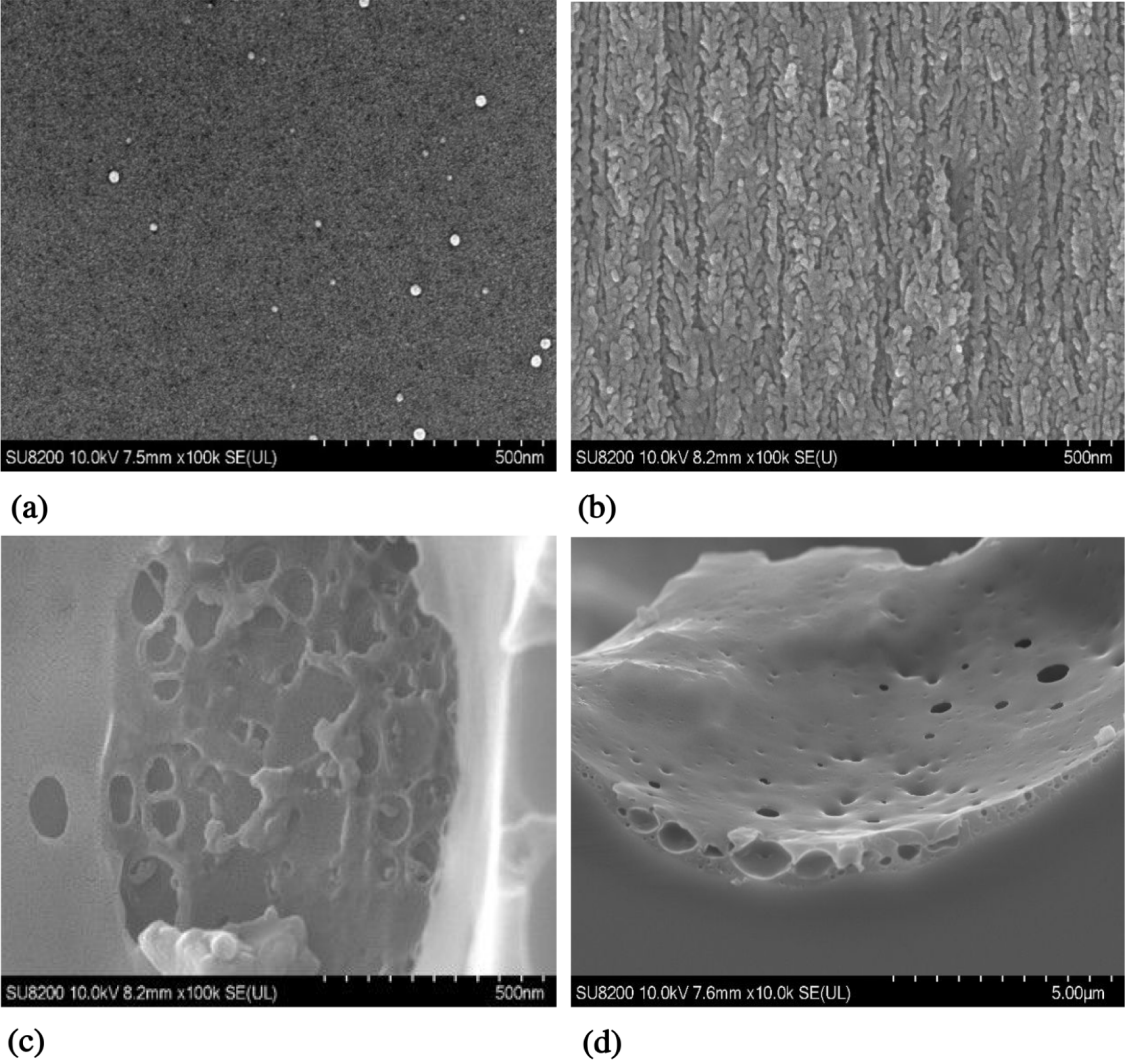
SEM images of silicon samples anodized at different temperatures, highlighting the evolution of porosity and etching anisotropy. All the images were acquired via a Schottky field-emission SEM (SU8030, Hitachi) at an accelerating voltage of 10.0 kV with a magnification of 100,000× and an SE (secondary electron) detector. (a, b) Anodization at room temperature (RT): The top view (a) shows scattered surface features, whereas the cross-sectional view (b) reveals highly anisotropic, vertically aligned mesopores. (c, d) Anodization at −70 °C: The top view (c) and cross-sectional view (d) display a nearly isotropic morphology characterized by circular nanopits and interconnected porous domains, indicating a transition to diffusion-limited, radially symmetric dissolution.

Although both cryogenic anodization and room-temperature anodization–oxidation-etching^52,53^ on heavily boron-doped silicon can produce silicon nanocrystals that exhibit visible PL, their structural components differ markedly. Cryogenic anodization enables the direct formation of 1.8–4.0 nm silicon nanocrystals with well-protected, passivated surfaces with hydrogen or fluorine termination. In contrast, oxidation–etching begins with larger porous silicon frameworks that are subsequently downsized via thermal oxidation and etching. This indirect pathway often compromises crystallinity because of disordered oxidation-induced disruption, resulting in a large decrease in the PL intensity.^54^

Our results show that these nanocrystals retain bright blue PL over one year of ambient storage without vacuum encapsulation. In contrast, oxidation-etched surfaces frequently exhibit trap states that induce the formation of oxides or Si–OH groups, which readily interact with moisture and oxygen, thereby accelerating PL degradation within hours. For applications requiring long-term stability, cryogenic anodization offers a more robust and reliable nanostructuring platform. To facilitate a direct comparison, the key differences in the anodization results between room temperature and cryogenic temperature are summarized in Table 1.

**Table 1:** Comparison of the Morphological, Structural, and Optical Characteristics of *p^++^*-type Silicon Anodized at Room Temperature versus Cryogenic Temperature

Anodization Feature	Room Temperature (RT, 25°C)	Cryogenic Temperature (−70 °C ∼ −80°C)
Etching Morphology (SEM)	Deep, vertical tubular channels (Anisotropic etching dominated)	Shallow, isotropic pit-like structures (Reaction-rate limited)
Pore Architecture	Continuous mesoporous network	Isolated, discrete nanocrystalline domains
Crystallite Size (TEM)	Large (14–25 nm);^44^ Bulk-like	Ultrasmall (1.8–2.2 nm)
Photoluminescence (PL)	None (Appears black under UV)	Strong Blue/Violet Emission
Dominant Etching Mechanism	Hole-supply imitated dissolution (Gösele–Lehmann model)	Fluorine-diffusion limited and surface passivation (*SiO*_*x*_ *F*_*y*_ shell formation)

### Cryogenic Control of Pore Morphology and Crystallite Size

In this study, we demonstrate that the morphological evolution of porous silicon is strictly governed by fluorination anodization, which is critically modulated by a sufficiently low electrolyte temperature. At room temperature, SEM and TEM results reveal that the dissolution kinetics are very aggressive for highly boron-doped silicon, leading to the formation of deep, irregular tubular channels with broad crystallite size distributions.^44^ Anodization of heavily doped *p*-type silicon at room temperature typically yields a continuous mesoporous network rather than the isolated quantum structures required for visible emission under UV irradiation. While the recent optimization of parameters such as the HF concentration and current density has been explored to modulate pore morphology, the electrolyte temperature has emerged as the decisive factor in scaling down feature sizes.^55,56^ By suppressing the anisotropic orientation of the Gösele–Lehmann-model etching process, cryogenic conditions facilitate a transition toward an isotropic reaction-limited oxidation–HF etching pathway, enabling the formation of blue-emissive nanocrystals. Mebed et al.^56^ demonstrated that lowering the electrolyte temperature to 0 °C reduced the pore diameter to approximately 10 nm, but this condition remains insufficient for accessing the sub3 nm quantum regime. By pushing the anodization temperature close to the cryogenic threshold (−78 °C), we successfully suppressed the sidewall dissolution kinetics, refining the crystallite size down to 1.8–2.2 nm (Figure 4). On the other hand, the origin of photoluminescence in silicon nanostructures remains a subject of ongoing debate, specifically regarding the competition between quantum confinement (QC) effects and surface defect states. Previous studies by Basu et al.^57^ and Tifouti et al.^58^ have established that while red/orange emission typically arises from trap-to-trap transitions at oxide interfaces, blue-shifted emission serves as a hallmark of band-to-band transitions involving quantum-confined Bloch states within ultrasmall silicon cores. In our study, the cryogenically anodized samples exhibited strong blue emission (∼415 nm and ∼500 nm) that correlates precisely with the bandgap expansion predicted for ∼2 nm silicon nanocrystals. The complete absence of red luminescence in these cryogenic samples, coupled with the structural evidence of a well-defined mesoporous framework, strongly suggests that the observed emission originates from the intrinsic quantum confinement of the silicon core rather than surface oxide defects. However, achieving blue luminescence from silicon typically requires complex postprocessing to obtain defect-free nanocrystals. For instance, the synthesis of blue-emitting silicon nanoparticles (∼447 nm) via quantum confinement involved a multistep process^59^ requiring the liftoff of a free-standing porous silicon layer followed by aggressive ultrasonic fragmentation. In contrast, cryogenic anodization provides a robust, one-step electrochemical route to fabricate blue-emitting nanocrystals directly on a silicon substrate. This approach not only simplifies the fabrication workflow but also maintains the structural integrity of the active layer, offering a significant advantage for scalable optoelectronic integration.

## Conclusion

In this work, we have demonstrated a direct, one-step synthesis of blue-luminescent silicon nanocrystals via cryogenic electrochemical anodization at −70 °C to −80 °C. By systematically investigating the impact of temperature, we determined that the cryogenic environment is the critical factor in suppressing anisotropic chemical dissolution—tentatively attributed to an in situ oxidation-driven pathway operating within the HF-based electrolyte. This mechanism enables the precise refinement of silicon crystallites from 14–16 nm (at room temperature) to the quantum-confined regime of 1.8–4.0 nm. The observed blue photoluminescence is attributed to strong quantum confinement effects in the ultrasmall silicon cores, which is consistent with the Bloch-state transition models and is distinct from the defect-induced red emission commonly found in larger structures. Beyond spectral control, this cryogenic fluorination process yields nanocrystals with high structural quality and sustained surface passivation, retaining bright photoluminescence for more than one year under ambient conditions without encapsulation. Consequently, these findings establish cryogenic anodization as a robust, CMOS-compatible approach for bandgap engineering, paving the way for the development of stable silicon-based blue light-emitting devices and future optoelectronic integration.

## Supplementary Material











## Notes

Videos S1–S4: Real-time visualization of bubble evolution during the electrochemical anodization process at −70 °C; Videos S1: the initial stages (5 min) at positions far from the cathode (MP4)
Videos S2: the initial stages (5 min) at positions close to the cathode (MP4)
Videos S3: the status after 45 min of processing at positions close to the cathode (MP4)
Video S4: the status after 45 min of processing at positions far from the cathode (MP4)
UV-illuminated photoluminescence photographs and spectra, SEM images of surface and cross-sectional morphologies, and specifications of the heavily boron-doped silicon wafers (PDF)

## Notes

During the preparation of this manuscript, the authors used OpenAI/ChatGPT and Google/Gemini for partial language translation and engaged American Journal Experts (LLC) in the Automated Language Editing Tool (Rubriq) to ensure adherence to academic communication standards. The authors have carefully reviewed and revised the content following these services and take full responsibility for the final version of the manuscript.
