# Supplementary material for: Altering the Fluorination Anodization Pathway of Silicon Near an Electrolyte Freezing Point Promotes the Formation of Blue-Photoluminescent Microstructures
Source: ACS Appl Mater Interfaces. 2026 Jan 29;18(5):9238–47. doi: 10.1021/acsami.5c24266 (PMC12903104; doi:10.1021/acsami.5c24266)
Supplement: Supplementary file 5 [file am5c24266_si_005.pdf]

Supporting Information

Altering the Fluorination Anodization Pathway of Silicon near an Electrolyte Freezing Point Promotes the Formation of Blue-Photoluminescent Microstructures

Yu-Sheng Chiou[1], Chao-Chia Cheng[2], Ryan Wen-Shuo Li[1], and Benjamin Tien-Hsi Lee[1,*]

[1] Department of Mechanical Engineering, National Central University, Taoyuan City 320317, Taiwan, Republic of China

[2] Department of Physics, National Central University, Taoyuan City 320317, Taiwan, Republic of China

*Corresponding author: Professor Benjamin Lee, Email: benlee@ncu.edu.tw

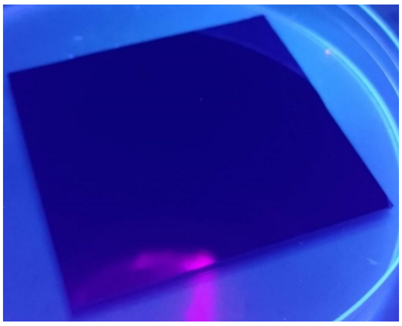

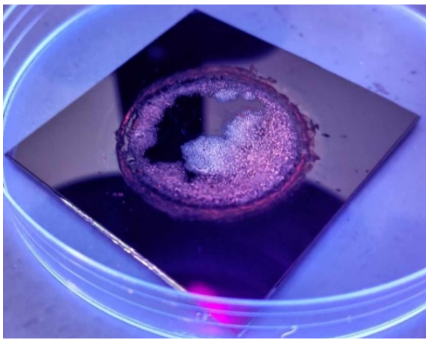

(a) (b)

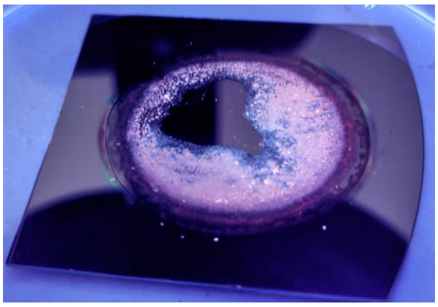

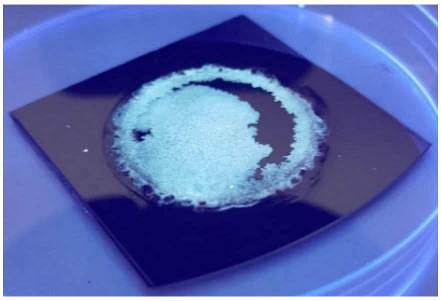

(c) (d)

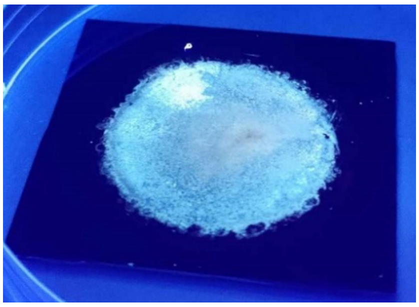

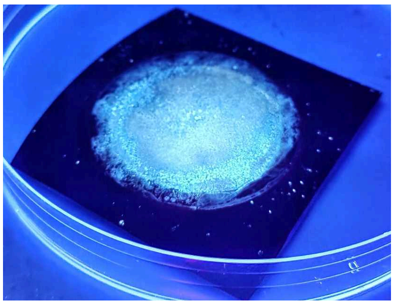

(e) (f)

Figure S1. Photoluminescence under UV illumination of heavily boron doped *p*$^+$-type silicon (0.003–0.004 Ω·cm) anodized at different temperatures: (a) room temperature (25°C), black, no PL; (b) -30°C, deep pink PL; (c) -40°C, pink–white PL; (d) -50°C, white PL; (e) -60°C, cyan PL; (f) -70°C, cyan-blue PL.

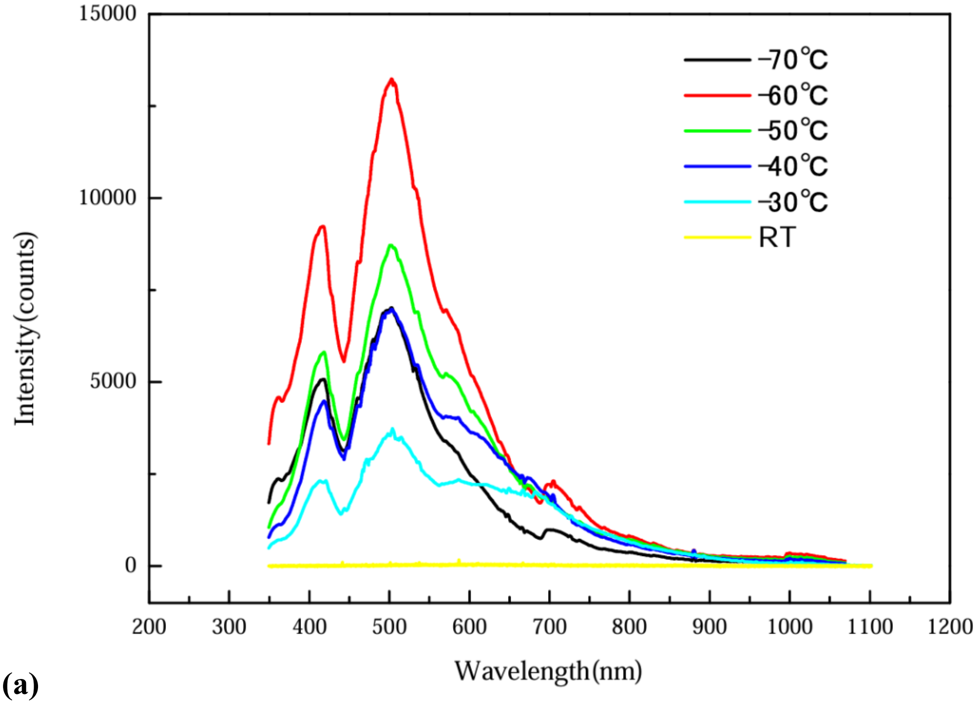

(a)

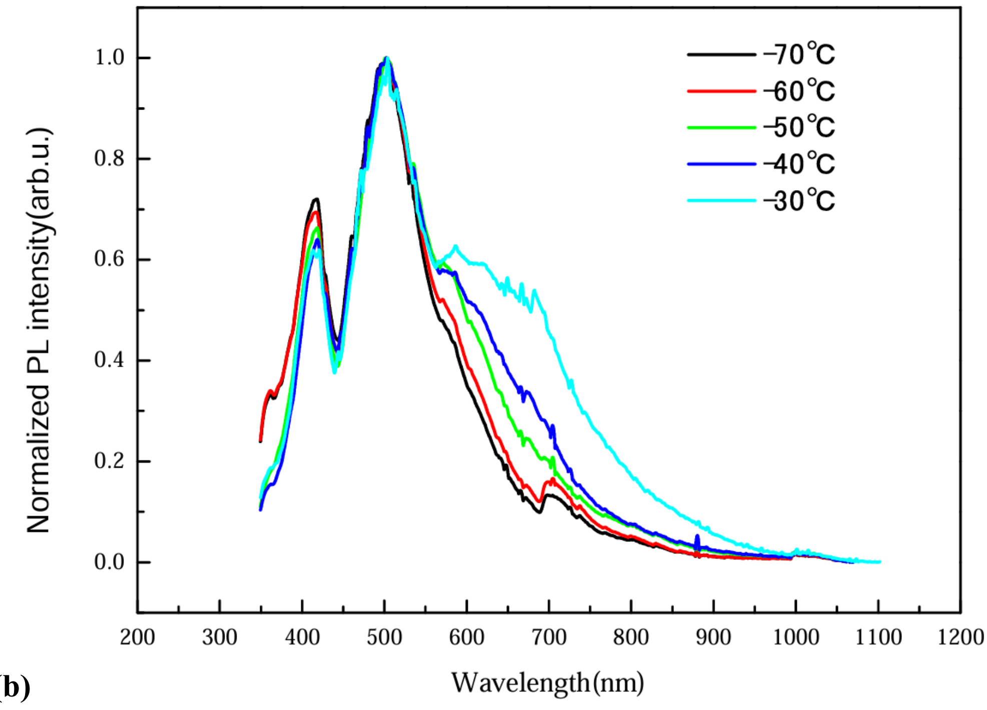

(b)

Figure S2. PL spectra of heavily doped p^+-type silicon anodized at different temperatures: (a) Comparison of PL intensity; (b) Comparison of normalized PL intensity, where starting from -30°C, as the temperature decreases, the energy of the red, orange, and yellow emission bands gradually decreases until the lowest at -70°C.

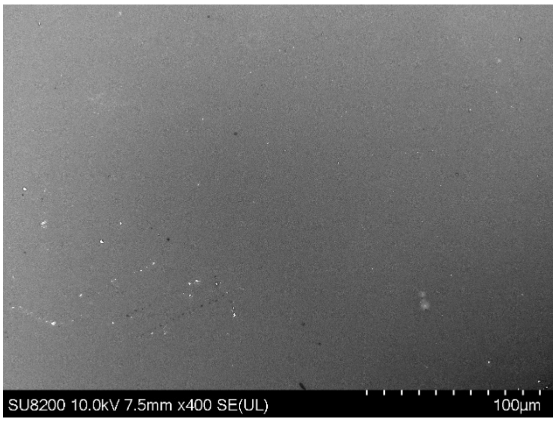

(a)

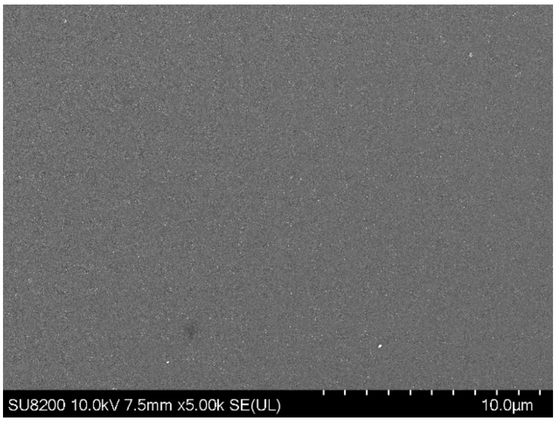

(b)

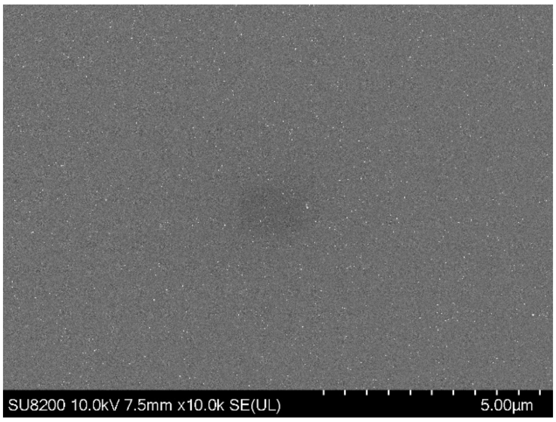

(c)

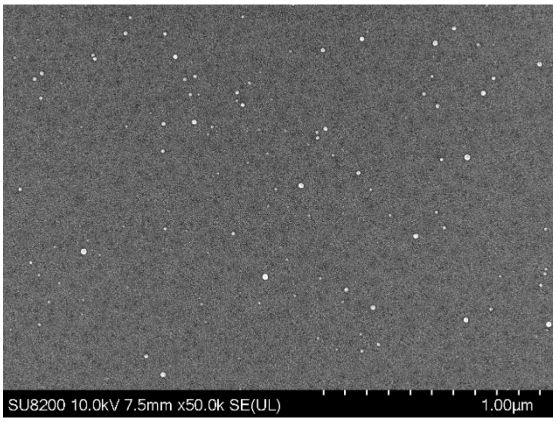

(d)

Figure S3. SEM top view images of p^+-type silicon anodized at room temperature: (a) 400X; (b) 5000X; (c) 10000X; (d) 50000X.

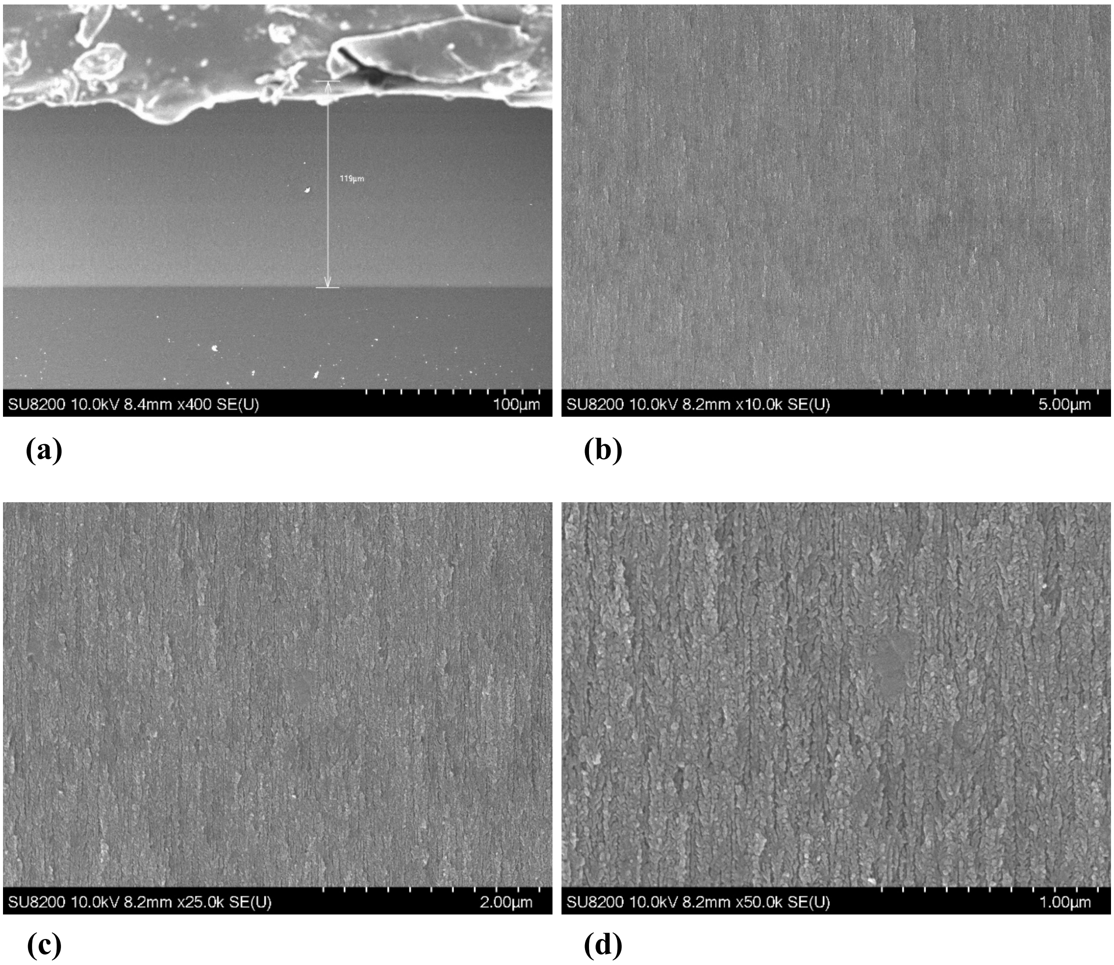

Figure S4. SEM cross-sectional view images of p^+-type silicon anodized at room temperature: (a) 400X; (b) 10000X; (c) 25000X; (d) 50000X.

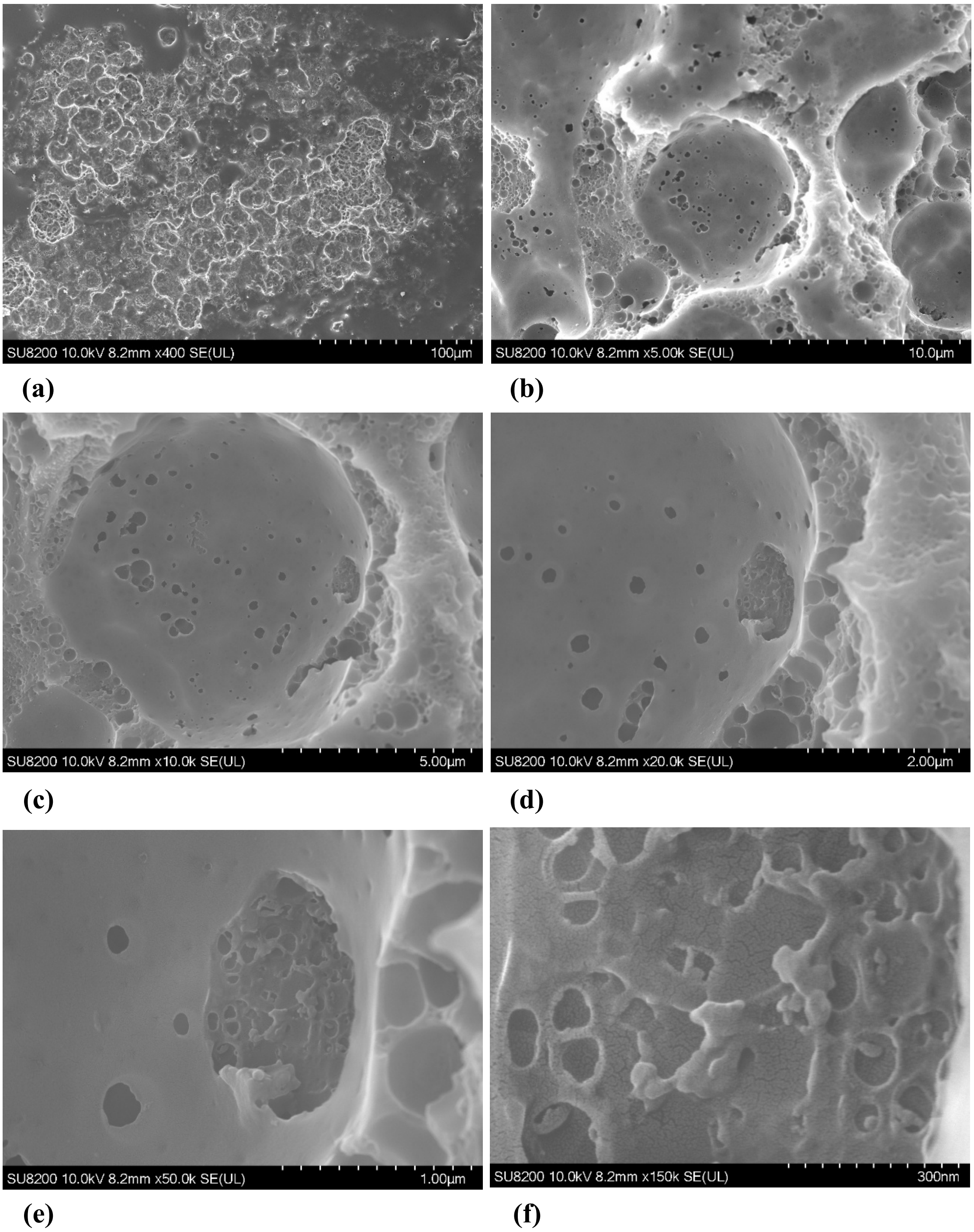

Figure S5. SEM top view images of p^+-type silicon anodized at cryogenic temperature (-70°C): (a) 400X; (b) 5000X; (c) 10000X; (d) 20000X; (e) 50000X; (f) 150000X.

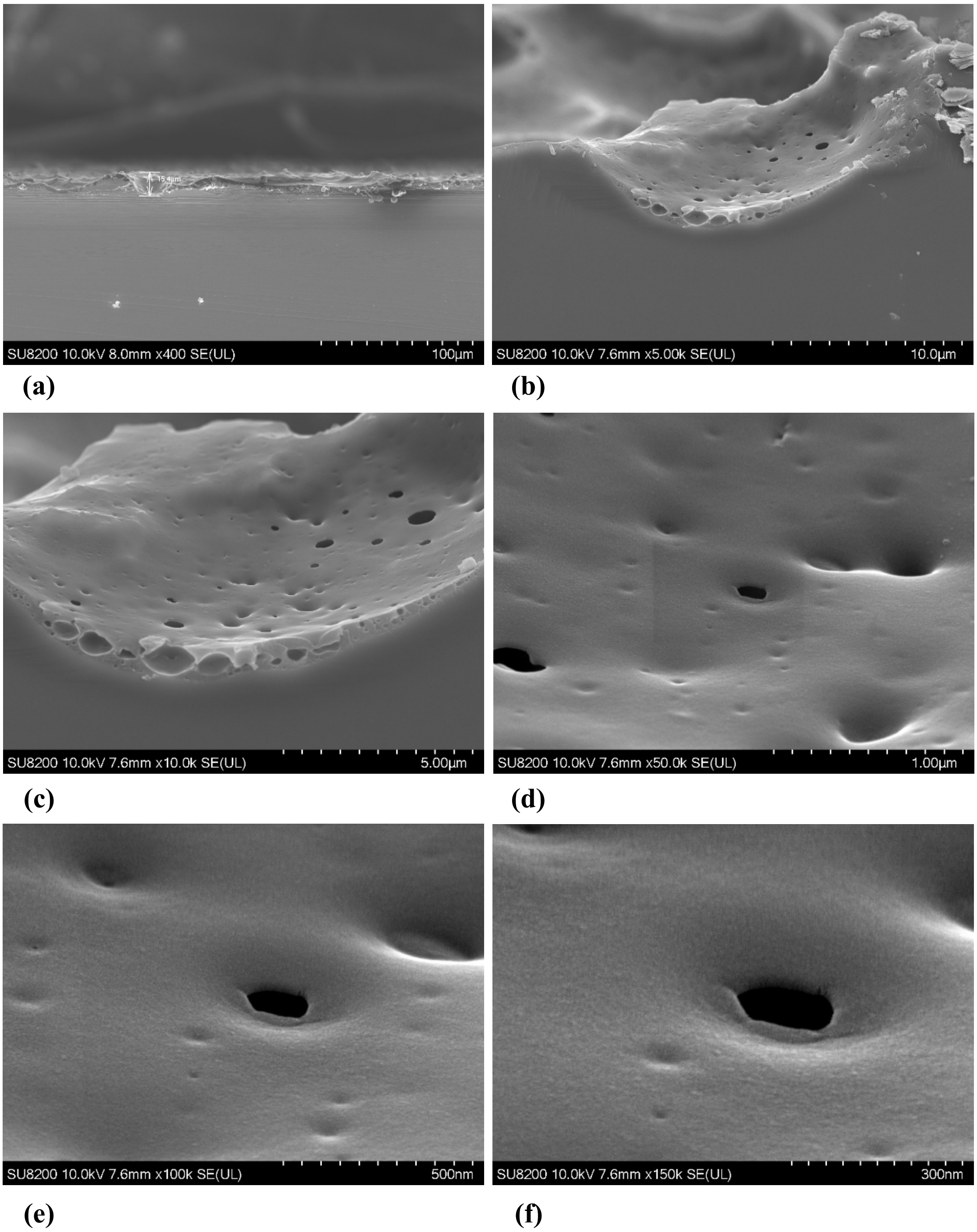

Figure S6. SEM cross-sectional view images of p^+-type silicon anodized at cryogenic temperature (-70°C): (a) 400X; (b) 5000X; (c) 10000X; (d) 50000X; (e) 100000X; (f) 150000X.

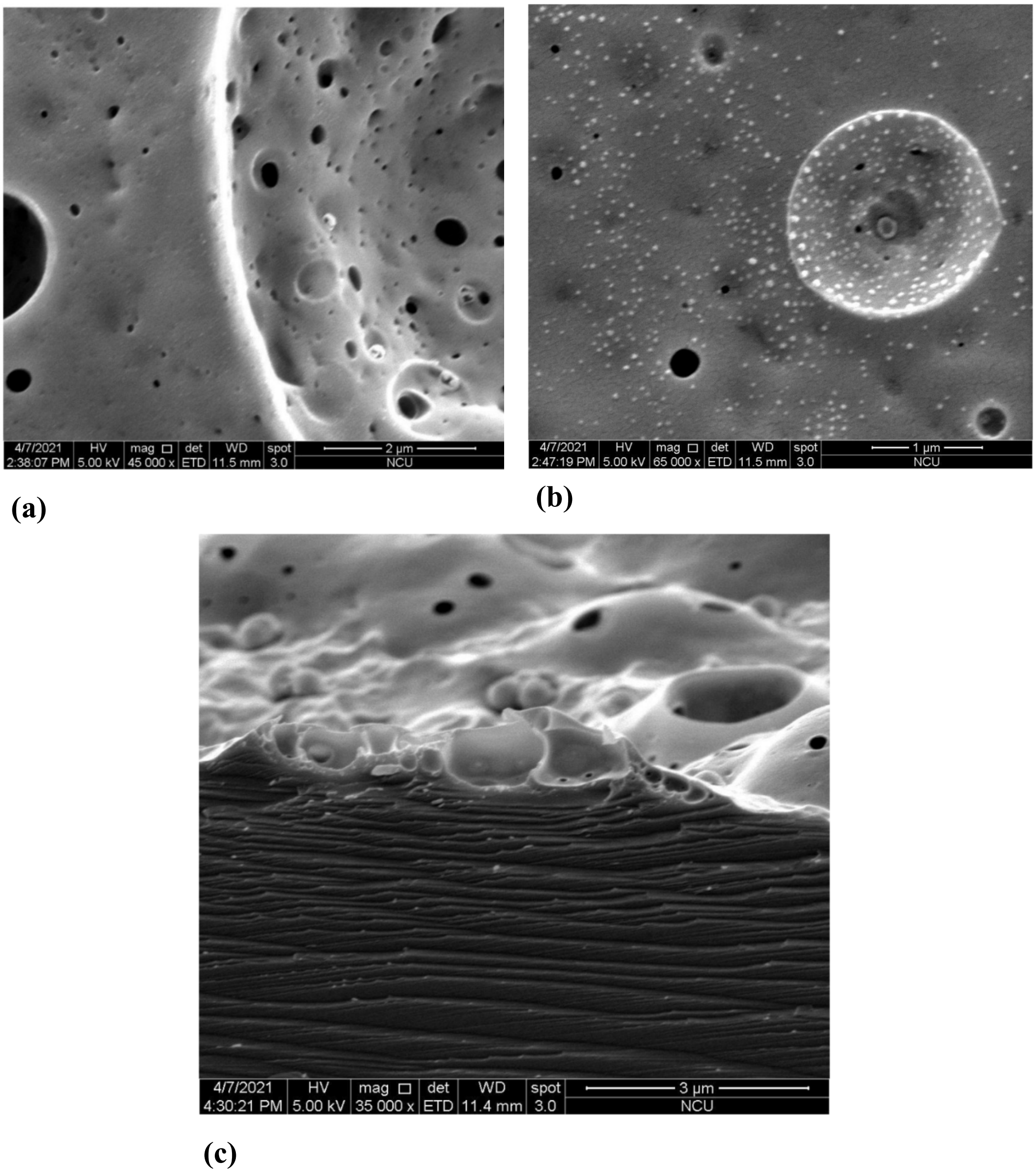

Figure S7. SEM images of p^+-type silicon anodized at a challenging cryogenic temperature (-80°C): (a) top view, 45000X; (b) top view, 65000X; (c) cross-sectional view, 35000X.

Supporting Tables

Table S1. Specifications of the heavily boron-doped silicon wafers used in this study.

DIAMETER (mm)	149.8-150.2	PRODUCT	Prime
METHOD	CZ	TYPE	P
ORIENTATION	1-0-0	DOPANT	Boron
THICKNESS (μm)	660-690	RESISTIVITY (Ω cm)	0.003-0.004
